# Supplementary material for: Nearest neighbor rules for RNA helix folding thermodynamics: improved end effects
Source: Nucleic Acids Res. 2022 May 7;50(9):5251–62. doi: 10.1093/nar/gkac261 (PMC9122537; doi:10.1093/nar/gkac261)
Supplement: gkac261_Supplemental_Files [file gkac261_supplemental_files.zip › Supplemental Information.pdf]

Supplemental Information to Accompany:

RNA Helix Folding Thermodynamics: Expanded Sequence Dependence of Ends Improves Nearest Neighbor Rules

Jeffrey Zuber*, Susan J. Schroeder*, Hongying Sun, Douglas H. Turner, David H. Mathews[1]
*These authors contributed equally.
[1]Corresponding author

Supplemental Table 1. Duplex sequences used for parameter fitting.

Sequence	ΔH° (kcal/mol)	ΔS° (eu)	ΔG° (37 °C) (kcal/mol)	Ref
**CAAAAAAG**<br>**GUUUUUUC**	-53.83	-158.7	-4.61	(33)
**CAAAAAAAG**<br>**GUUUUUUUC**	-59.78	-175.1	-5.47	(33)
**CCGG/**	-34.21	-95.6	-4.56	(34)
**ACCGGU/**	-59.5	-164.5	-8.48	(34)
**CCGG/**	-31	-86.5	-4.17	(34)
**GGCC/**	-35.79	-98.1	-5.36	(35)
**GGCC/**	-41.3	-116	-5.32	(35)
**AGGCCU/**	-52	-140	-8.58	(36)
**AGGCCU/**	-48.19	-128.4	-8.37	(36)
**CGGCCG/**	-54.12	-142.57	-9.90	(36)
**UGGCCA/**	-59.9	-164	-9.04	(36)
**GCCGGC/**	-62.72	-166	-11.24	(36)
**UCCGGA/**	-51.92	-142.3	-7.79	(36)
**GCGC/**	-30.48	-83.4	-4.61	(37)
**GCGCGC/**	-65.98	-178.5	-10.62	(37)
**CGCGCG/**	-54.51	-146.4	-9.10	(37)
**UGGCCG/**	-53	-143.3	-8.56	(38)
**UCCGGG/**	-47.7	-129.8	-7.44	(38)

**GCCGGU/**	-58.2	-158.1	-9.17	(38)
**GGCGCU/**	-56.4	-154.7	-8.42	(38)
**CGCG/**	-31.8	-91.6	-3.39	(39)
**UGCGCA/**	-51.54	-139.7	-8.21	(39)
**AGCGCU/**	-50.07	-135.7	-7.98	(39)
**GGCGCC/**	-67.78	-182	-11.33	(39)
**AUGCGCGU/**	-54.9	-147	-9.31	(40)
**AUGCGUAU/**	-46.8	-133.9	-5.27	(40)
**AUGUGCAU/**	-57.1	-164.2	-6.17	(40)
**GCUGGC/**	-59.1	-169.7	-6.47	(40)
**GGCGUC/**	-38.1	-107.8	-4.67	(40)
**CUGCGG/**	-41.4	-119.6	-4.31	(40)
**CGGCUG/**	-43.2	-121.4	-5.55	(40)
**AUGCAU/**	-41.7	-119.2	-4.73	(40)
**AUGCGCAU/**	-64.39	-174.8	-10.18	(40)
**AUACGUAU/**	-54.36	-154.2	-6.53	(40)
**AUGUACAU/**	-55.91	-159.3	-6.50	(40)
**GCUAGC/**	-59.13	-165.1	-7.92	(40)
**GACGUC/**	-58.06	-163.5	-7.35	(40)
**CUGCAG/**	-55.41	-155.7	-7.12	(40)
**CAGCUG/**	-51.55	-144.7	-6.67	(40)

**AGAUAUCU/**	-64.51	-186.8	-6.57	(41)
**AUCUAGAU/**	-59.89	-169.9	-7.20	(41)
**AACUAGUU/**	-54.62	-153	-7.17	(41)
**AGUUAACU/**	-52.42	-148.5	-6.36	(41)
**ACUUAAGU/**	-47.23	-132.4	-6.17	(41)
**GAACGUUC/**	-77	-218.3	-9.29	(41)
**GUUCGAAC/**	-74.19	-211	-8.75	(41)
**UCUAUAGA/**	-62.06	-177.7	-6.95	(41)
**UAGAUCUA/**	-60.15	-170.6	-7.24	(41)
**GUCGAC/**	-53.63	-150.1	-7.08	(41)
**ACUAUAGU/**	-59.21	-168.4	-6.98	(41)
**UGAUCA/**	-44.73	-128	-5.03	(41)
**GCAUGC/**	-62.34	-177.2	-7.38	(42)
**GUGCAC/**	-59.61	-167.5	-7.66	(42)
**GUCUAGAC/**	-76.02	-212.5	-10.11	(42)
**GAUAUAUC/**	-62.04	-180.4	-6.09	(42)
**GUAUAUAC/**	-63.35	-185.1	-5.94	(42)
**GAGAGA<br>CUCUCU**	-62.05	-178.1	-6.81	(42)
**AGAGAGAG<br>UCUCUCUC**	-73.66	-201.7	-11.10	(42)
**AAUGCAUU/**	-59.81	-169.7	-7.18	(43)
**CAUGCAUG/**	-73.67	-206.3	-9.69	(43)

**GAUGCAUC/**	-72.75	-201.9	-10.13	(43)
**UAUGCAUA/**	-67.73	-195	-7.25	(43)
**UGCGCA/**	-50.8	-137.6	-8.12	(43)
**GAUGCAUU/**	-62.9	-180.8	-6.82	(44)
**UAUGCAUG/**	-62.3	-180.1	-6.44	(44)
**GCGGCG**<br>**CGCCGC**	-58.5	-155	-10.43	(45)
**UGACCUCA**<br>**ACUGGAGU**	-76.09	-205.55	-12.34	(46)
**CGCGCG/**	-54.74	-146.77	-9.22	(46)
**CACAG**<br>**GUGUC**	-40.2	-115.4	-4.41	(47)
**AUGGUCAU/**	-57.4	-167.6	-5.42	(48)
**CCUGUAGG/**	-71.1	-207.3	-6.81	(48)
**CGGGUCCG/**	-81.4	-226.4	-11.18	(48)
**CGUUGACG/**	-73.5	-214.6	-6.94	(48)
**CUGGUCAG/**	-70.5	-204.4	-7.11	(48)
**GGAGUUCC/**	-73.1	-214.9	-6.45	(48)
**GGAUGUCC/**	-73	-208.4	-8.36	(48)
**GGCGUGCC/**	-73.4	-206.9	-9.23	(48)
**GUCGUGAC/**	-69.1	-203.3	-6.05	(48)
**GAGUUGAG**<br>**CUCGGCUC**	-73.9	-211.8	-8.21	(48)
**AGUCGAUU/**	-53.3	-152.6	-5.97	(48)
**CGGAUUCG/**	-72.6	-213	-6.54	(48)

**CCUAGG/**	-54.1	-149.1	-7.86	(48)
**CGUACG/**	-46.6	-133.1	-5.32	(48)
**GGAUCC/**	-53.7	-149.1	-7.46	(48)
**GAGCUC/**	-54.85	-151.94	-7.73	(49)
**GCGUGC/**	-46.18	-132.43	-5.11	(49)
**GAGGUCUC/**	-82.8	-238.7	-8.77	(50)
**GAGCUC/**	-62.3	-175.3	-7.93	(50)
**GGUACC/**	-54.9	-153.4	-7.32	(50)
**GAGUGCUC/**	-83	-237.4	-9.37	(51)
**GGUUGACC/**	-78.3	-225.9	-8.24	(51)
**GGCUGGCC/**	-87.2	-238.8	-13.14	(51)
**CCAUGUGG/**	-70.5	-202.1	-7.82	(52)
**CCAGUUGG/**	-61.1	-178.6	-5.71	(52)
**CCAUGG/**	-56.93	-159.9	-7.34	(52)
**GAGGAG**<br>**CUCCUC**	-55.7	-152.2	-8.50	(53)
**GAGUGGAG**<br>**CUCGUCUC**	-82.3	-234.1	-9.69	(53)
**GAGUUGAG**<br>**CUCGGCUC**	-70	-199.2	-8.22	(53)
**GAGGUGAG**<br>**CUCUGCUC**	-78.4	-228.2	-7.62	(53)
**CGCG/**	-33.31	-95.6	-3.66	(53)
**ACGCA**<br>**UGCGU**	-45.4	-130.4	-4.96	(53)
**AGCGA**<br>**UCGCU**	-46.31	-133	-5.06	(53)

**GCACG**<br>**CGUGC**	-45.31	-126.2	-6.17	(53)
**GCUCG**<br>**CGAGC**	-43.38	-120.1	-6.13	(53)
**CACGUG/**	-50.31	-141	-6.58	(53)
**CCGCGG/**	-60.79	-164.3	-9.83	(53)
**GCAACG**<br>**CGUUGC**	-50.57	-140.5	-6.99	(53)
**GCAUCG**<br>**CGUAGC**	-51.89	-143.9	-7.26	(53)
**GCCGCG**<br>**CGGCGC**	-59.69	-157.4	-10.87	(53)
**GCGCCG**<br>**CGCGGC**	-57.85	-151.3	-10.92	(53)
**GCGCGG**<br>**CGCGCC**	-71.16	-192.7	-11.39	(53)
**GCGUCG**<br>**CGCAGC**	-52.38	-140.6	-8.77	(53)
**GCUACG**<br>**CGAUGC**	-58.02	-162.7	-7.56	(53)
**GGCGCG**<br>**CCGCGC**	-63.53	-170.1	-10.77	(53)
**GUGGUG**<br>**CACCAC**	-48.84	-132.7	-7.68	(53)
**GUGUCG**<br>**CACAGC**	-50.88	-140.9	-7.18	(53)
**UCAUGA/**	-41.89	-121.2	-4.30	(53)
**UCGCGA/**	-48.94	-135.7	-6.85	(53)
**UCUAGA/**	-36.53	-101.8	-4.96	(53)
**AAGGAGG**<br>**UUCCUCC**	-58.72	-158.6	-9.53	(53)
**ACUGUCA**<br>**UGACAGU**	-52.24	-142.9	-7.92	(53)
**AGUCUGA**<br>**UCAGACU**	-51.48	-141.8	-7.50	(53)
**GACUCAG**<br>**CUGAGUC**	-64.11	-177.5	-9.06	(53)

**GAGUGAG**<br>**CUCACUC**	-70.49	-196	-9.70	(53)
**GUCACUG**<br>**CAGUGAC**	-57.81	-158.6	-8.62	(53)
**ACCUUUGC**<br>**UGGAAACG**	-77.42	-215.3	-10.64	(53)
**CGACGCAG**<br>**GCUGCGUC**	-70.45	-187.43	-12.32	(53)
**CUCGCACA**<br>**GAGCGUGU**	-72.61	-195.09	-12.10	(53)
**GGCUUCAA**<br>**CCGAAGUU**	-61.59	-165.7	-10.20	(53)
**UCCUUGCA**<br>**AGGAACGU**	-70.27	-190.82	-11.09	(53)
**UUCCGGAA/**	-67.43	-182.6	-10.80	(53)
**UUGCGCAA/**	-62.17	-167.6	-10.19	(53)
**UUGGCCAA/**	-63.68	-169.8	-11.02	(53)
**UUGUACAA/**	-49.45	-137.8	-6.71	(53)
**AAGGUUGGAA**<br>**UUCCAACCUU**	-75.84	-203.62	-12.69	(53)
**CAUGCG**<br>**GUACGC**	-48.57	-134	-7.01	(53)
**GAGCUG**<br>**CUCGAC**	-51.59	-142.2	-7.49	(53)
**GCUGAG**<br>**CGACUC**	-55.85	-155.2	-7.71	(53)
**GUGCAG**<br>**CACGUC**	-55.94	-155.6	-7.68	(53)
**UAAGGUA**<br>**AUUCCAU**	-51.27	-142.9	-6.95	(53)
**GAGAUCUC/**	-75	-209.2	-10.12	(53)
**GCCAUGGC/**	-93.91	-254.2	-15.07	(53)
**GCUGCGAC**<br>**CGACGCUG**	-86.18	-232.95	-13.93	(53)
**UCCGCGCA**<br>**AGGCGCGU**	-81.15	-214.61	-14.59	(53)

**GAGUGAG**<br>**CUCGCUC**	-64.2	-181.8	-7.81	(54)
**GCGGCG**<br>**CGCCGC**	-61.5	-163.1	-10.91	(54)
**GCGCGC/**	-68.2	-185	-10.82	(54)
**AAGGCCGGAA**<br>**UUCCGGCCUU**	-98.34	-258.54	-18.15	(28)
**CCUCUGGUGA**<br>**GGAGACCGCU**	-93.46	-251.96	-15.31	(28)
**AGGCCGGA**<br>**UCCGGCCU**	-84.09	-221.91	-15.26	(28)
**GAGCCGAC**<br>**CUCGGCUG**	-80.13	-213.99	-13.76	(28)
**UCACCUGA**<br>**AGUGGACU**	-71.51	-195.62	-10.84	(28)
**CUGGUC**<br>**GACCAG**	-55.1	-151.7	-8.05	(55)
**CCAGCGUCCU**<br>**GGUUGUAGGA**	-87.9	-246	-11.60	(56)
**GAGGAC**<br>**CUCCUG**	-57.3	-157.1	-8.58	(56)
**GCAUGUGC/**	-72.4	-206.1	-8.48	(57)
**GCAGUUGC/**	-64.8	-190	-5.87	(57)
**GCUGGUGC**<br>**CGAUUACG**	-69.4	-199.1	-7.65	(57)
**GUCUAGAU/**	-70	-201	-7.66	(58)
**AUCUAGAU/**	-59.1	-166.9	-7.34	(58)
**GGCAUGCC/**	-90.2	-244.1	-14.49	(59)
**GGCUAGCC/**	-76.2	-203	-13.24	(59)
**GGCAAGGC**<br>**CCGUUCCG**	-72.8	-192.6	-13.07	(59)
**GGCGGGGC**<br>**CCGUUCCG**	-76.5	-202.2	-13.79	(59)
**UCCGCC**<br>**AGGUGG**	-57	-162.2	-6.69	(60)

**GAGUUGAC**<br>**CUCAACUG**	-88.7	-251.8	-10.60	(60)
**GUUGCAGU/**	-45.2	-128.3	-5.41	(61)
**UGCGAUCCUG/**	-69.5	-201.6	-6.97	(61)
**AGCGCU/**	-49.5	-134.5	-7.78	(61)
**UUGCAG/**	-37.2	-106.5	-4.17	(61)
**GUGCAU/**	-47.5	-136.9	-5.04	(61)
**UUUGCAGG/**	-46.1	-133.1	-4.82	(61)
**UUAUCGAUGG/**	-61.1	-175	-6.82	(61)
**GGAUGCAUUU/**	-74.4	-214.2	-7.97	(61)
**UGAUGCAUUG/**	-75.4	-216.9	-8.13	(61)
**GUAUCGAUGU/**	-63.4	-180.2	-7.51	(61)
**GUCGCGGU/**	-57.8	-160.5	-8.02	(61)
**GUGAUCGU/**	-47.1	-132.5	-6.01	(61)
**UGUCGAUG/**	-68.8	-201.4	-6.34	(61)
**GGCAUGUU/**	-69.1	-201	-6.76	(61)
**UGCUAGUG/**	-74.9	-217.7	-7.38	(25)
**GGUCGCGGUU/**	-66.5	-186	-8.81	(25)
**GUGAUGCAUUGU/**	-93.7	-270.9	-9.68	(25)
**UGUGGCCGUG/**	-66.2	-178.3	-10.90	(25)
**UGUCGGUG/**	-57.3	-173.5	-3.49	(25)
**UUGGCUGG/**	-49.1	-141.7	-5.15	(25)

**GGUGGCCGUU/**	-66.4	-178.6	-11.01	(25)
**UUGCUAGUGG/**	-71.9	-206.5	-7.85	(25)
**GUGGCUGU/**	-52.3	-149.1	-6.06	(25)
**GUGCUAGUGU/**	-74	-211.3	-8.47	(25)
**UUGUCGGUGG/**	-64	-195.6	-3.33	(25)
**GUGUCGGUGU/**	-65.3	-197.5	-4.05	(25)
**UGUGGCUGUG/**	-55.5	-159.8	-5.94	(25)
**UAUCGAUA/**	-61.8	-176.2	-7.15	(25)
**UUAUCGAUAA/**	-82.3	-235.8	-9.17	(25)
**GCGGGAC**<br>**CGCUCUG**	-45.2	-116.8	-8.97	(24)
**CAGGGCUC**<br>**GUCUCGAG**	-62.8	-166.6	-11.13	(24)
**CUGGCUAG/**	-60.38	-171.8	-7.10	(24)
**GACGCCAG**<br>**CUGUGGUC**	-63.8	-171.8	-10.52	(24)
**GACGCGUU/**	-62.2	-169.9	-9.51	(24)
**GCAGCUGU/**	-72.3	-199.8	-10.33	(24)
**GGAGCUCU/**	-66.57	-180.9	-10.46	(24)
**GUAGCUAU/**	-50.3	-138.7	-7.28	(24)
**GUCGGGCC**<br>**CAGCUCGG**	-96	-261.3	-14.96	(24)
**UACCGGUG/**	-51.7	-135.4	-9.71	(24)
**UCACGUGG/**	-46.9	-124.2	-8.38	(24)
**UGACGUCG/**	-63.8	-172.3	-10.36	(24)

**UUACGUAG/**	-44.6	-124	-6.14	(24)
**CAGAGGAGAC GUCUUUUCUG**	-98.95	-288.6	-9.44	(24)
**CAGCGCGUUG/**	-77.02	-208.6	-12.32	(24)
**CAGUCGAUUG/**	-92.33	-269.7	-8.68	(24)
**CCGAAUUUGG/**	-76.76	-225	-6.98	(24)
**CGGAAUUUCG/**	-90.52	-266.5	-7.87	(24)
**CGGAUAUUCG/**	-88.2	-256.2	-8.74	(24)
**CGGGCGUUCG/**	-101.66	-290.5	-11.56	(24)
**CGGUGCAUCG/**	-102.42	-282.6	-14.77	(24)
**CUGGAUUCAG/**	-97.81	-282.7	-10.13	(24)
**GAGAGCUUUC/**	-86.57	-250.6	-8.85	(24)
**GAGGAUCUUC/**	-93.86	-270.9	-9.84	(24)
**GAGUGGAGAG CUCAUUUCUC**	-96.93	-280.7	-9.87	(24)
**GGUUCGGGCC/**	-115.71	-329.3	-13.58	(24)
**GUGAAUUUAC/**	-62.63	-186.4	-4.82	(24)
**GUGUGCAUAC/**	-58.6	-160.1	-8.94	(24)
**GUUAGCUGAC/**	-69.6	-196.7	-8.59	(24)

Supplemental Table 2

A)WCF helical parameters annotated with uncertainties estimated as standard errors of regression. These uncertainty estimates are not as good as those in Table 1 derived by perturbing the input experimental data.

	New Model		*1998 Model †*	
Feature	ΔG°_{37} (kcal/mol)	ΔH° (kcal/mol)	ΔG°_{37} (kcal/mol)	ΔH° (kcal/mol)
GC/CG	-3.46 ± 0.07	-16.52 ± 0.84	-3.42 ± 0.08	-14.88 ± 1.58
CC/GG	-3.28 ± 0.05	-13.94 ± 0.66	-3.26 ± 0.07	-13.39 ± 1.24
GA/CU	-2.42 ± 0.05	-13.75 ± 0.64	-2.35 ± 0.06	-12.44 ± 1.20
CG/GC	-2.33 ± 0.08	-9.61 ± 1.00	-2.36 ± 0.09	-10.64 ± 1.65
AC/UG	-2.25 ± 0.06	-11.98 ± 0.76	-2.24 ± 0.06	-11.40 ± 1.23
CA/GU	-2.07 ± 0.06	-10.47 ± 0.76	-2.11 ± 0.07	-10.44 ± 1.28
AG/UC	-2.01 ± 0.06	-9.34 ± 0.78	-2.08 ± 0.06	-10.48 ± 1.24
UA/AU	-1.29 ± 0.09	-9.16 ± 1.10	-1.33 ± 0.09	-7.69 ± 2.02
AU/UA	-1.09 ± 0.08	-8.91 ± 1.01	-1.10 ± 0.08	-9.38 ± 1.68
AA/UU	-0.94 ± 0.04	-7.44 ± 0.52	-0.93 ± 0.03	-6.82 ± 0.79
Initiation	+4.10 ± 0.20	+4.66 ± 2.47	+4.09 ± 0.22	+3.61 ± 4.12
AU End on AU	+0.22 ± 0.06	+4.36 ± 0.76	+0.45 ± 0.04 ‡	+3.72 ± 0.83 ‡
AU End on CG	+0.44 ± 0.04	+3.17 ± 0.55	+0.45 ± 0.04 ‡	+3.72 ± 0.83 ‡

B) GU stack parameters annotated with uncertainties estimated as standard errors of regression. These uncertainty estimates are not as good as those in Table 1 derived by perturbing the input experimental data.

	New Model		*2012 Model ¶*	
Feature	ΔG°_{37} (kcal/mol)	ΔH° (kcal/mol)	ΔG°_{37} (kcal/mol)	ΔH° (kcal/mol)
GC/UG	-2.23 ± 0.12	-14.73 ± 1.32	-2.15 ± 0.10	-11.09 ± 1.78
CU/GG	-1.93 ± 0.13	-9.26 ± 1.45	-1.77 ± 0.09	-9.44 ± 1.76
GG/CU	-1.80 ± 0.11	-12.41 ± 1.28	-1.80 ± 0.09	-7.03 ± 1.75
CG/GU	-1.05 ± 0.12	-5.64 ± 1.37	-1.25 ± 0.09	-5.56 ± 1.68
AU/UG	-0.76 ± 0.13	-9.23 ± 1.50	-0.90 ± 0.08	-7.39 ± 1.65
GA/UU	-0.60 ± 0.11	-10.58 ± 1.26	-0.51 ± 0.08	-10.38 ± 1.79
UG/GU	-0.38 ± 0.15	-8.76 ± 1.75	-0.57 ± 0.19	-12.64 ± 4.01
UA/GU	-0.22 ± 0.13	-2.72 ± 1.47	-0.39 ± 0.09	-0.96 ± 1.80
GG/UU	-0.20 ± 0.15	-9.06 ± 1.71	-0.25 ± 0.16	-17.82 ± 3.75
GU/UG	-0.19 ± 0.16	-7.66 ± 1.85	+0.72 ± 0.19	-13.83 ± 4.21
AG/UU	+0.02 ± 0.12	-5.10 ± 1.38	-0.35 ± 0.08	-3.96 ± 1.73
GGUC/CUGG	(-3.79 ± 0.27)†	(-32.48 ± 3.16)†	-4.12 ± 0.54	-30.80 ± 8.87
AU End on GU	-0.71 ± 0.30	5.16 ± 3.42	+0.45 ± 0.04*	+3.72 ± 0.83*
GU End on CG	0.13 ± 0.14	3.91 ± 1.63	0.00 ± 0.00 ‡	0.00 ± 0.00 ‡
GU End on AU	-0.31 ± 0.14	3.65 ± 1.56	0.00 ± 0.00 ‡	0.00 ± 0.00 ‡
GU End on GU	-0.74 ± 0.16	6.23 ± 1.89	0.00 ± 0.00 ‡	0.00 ± 0.00 ‡

Supplemental Table 3: Comparison of predictions of models with and without 5’GGUC/3’CUGG parameter.

	Model without 5’GGUC/3’GGUC[§]		Model with 5’GGUC/3’GGUC[§]		2012 Model[¶]	
**Duplex**	**ΔG°_{37} (kcal/mol)**	**ΔH° (kcal/mol)**	**ΔG°_{37} (kcal/mol)**	**ΔH° (kcal/mol)**	**ΔG°_{37} (kcal/mol)**	**ΔH° (kcal/mol)**
**AUGGUCAU UACUGGUA**	-5.14 ± 0.18	-57.86 ± 3.88	-5.50 ± 0.21	-62.57 ± 5.28	-5.12 ± 1.23	-59.39 ± 5.31
**CGGGUCCG GCCUGGGC**	-10.48 ± 0.15	-74.92 ± 2.98	-10.82 ± 0.20	-79.41 ± 5.06	-10.84 ± 1.18	-75.25 ± 4.95
**CUGGUCAG GACUGGUC**	-7.42 ± 0.14	-67.44 ± 3.16	-7.78 ± 0.17	-71.97 ± 4.89	-7.98 ± 1.13	-69.03 ± 4.80
**GAGGUCUC CUCUGGAG**	-8.12 ± 0.14	-74.00 ± 2.89	-8.48 ± 0.18	-78.57 ± 4.90	-8.46 ± 1.11	-73.03 ± 4.77

[§]Uncertainty values were calculated from the experiment covariation analysis. [¶]Uncertainty values were derived from standard errors of regression (24)

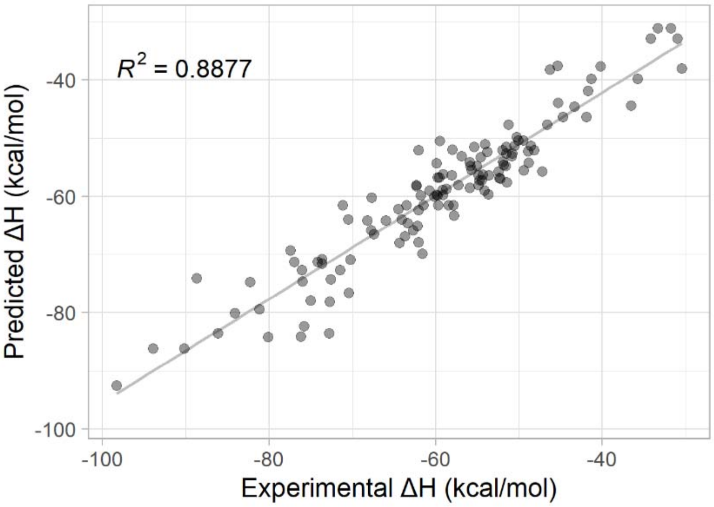

Supplemental Figure 1: ΔH° predictions as a function of experimentally-determine ΔH° for canonical WCF nearest neighbor parameters. ΔH° values predicted from updated nearest neighbor parameters for duplexes composed solely canonical WCF base pairs in Table 1A plotted against values determined from optical melting experiments.

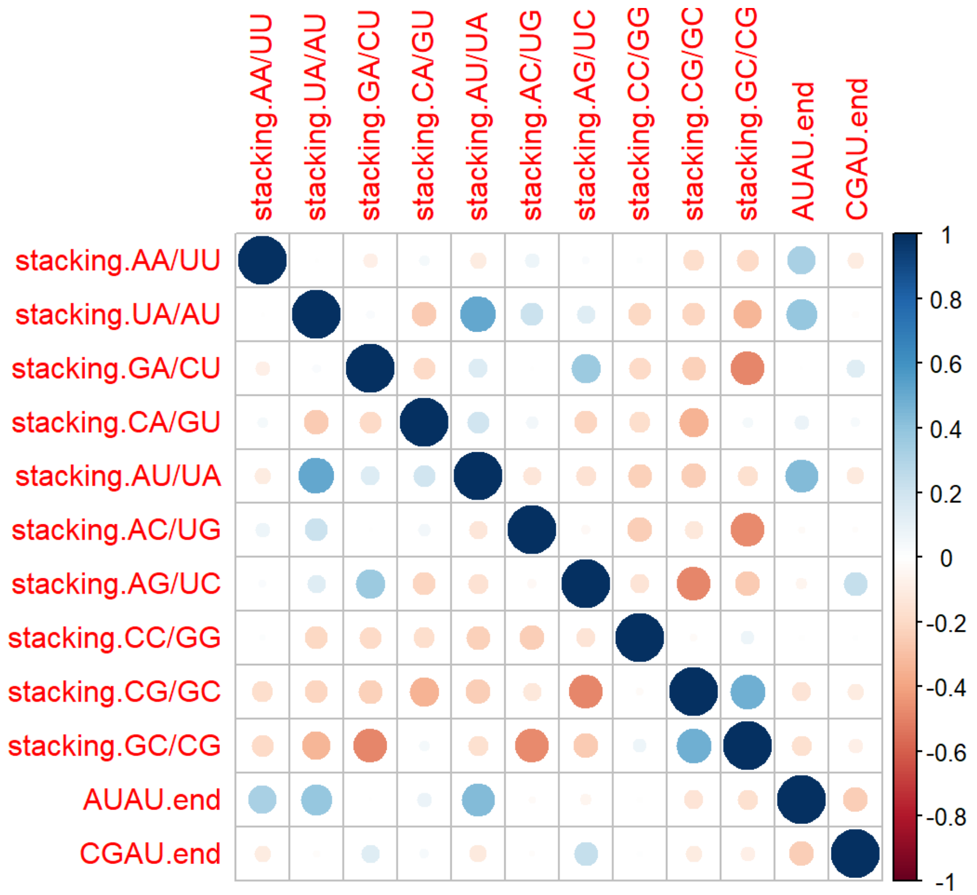

Supplemental Figure 2: Pearson correlations between model features. Pairwise Pearson correlation coefficients were calculated for the observed frequencies of WCF stacking parameters. The correlation coefficients are encoded in the size and color of each circle. The base pair stacks are represented a top strand (written 5' to 3') that is followed by a bottom strand (written 3' to 5'). Therefore, "AG/UC" indicates an AU pair followed by a GC pair: $\frac{5'\mathrm{AG}}{3'\mathrm{UC}}$.

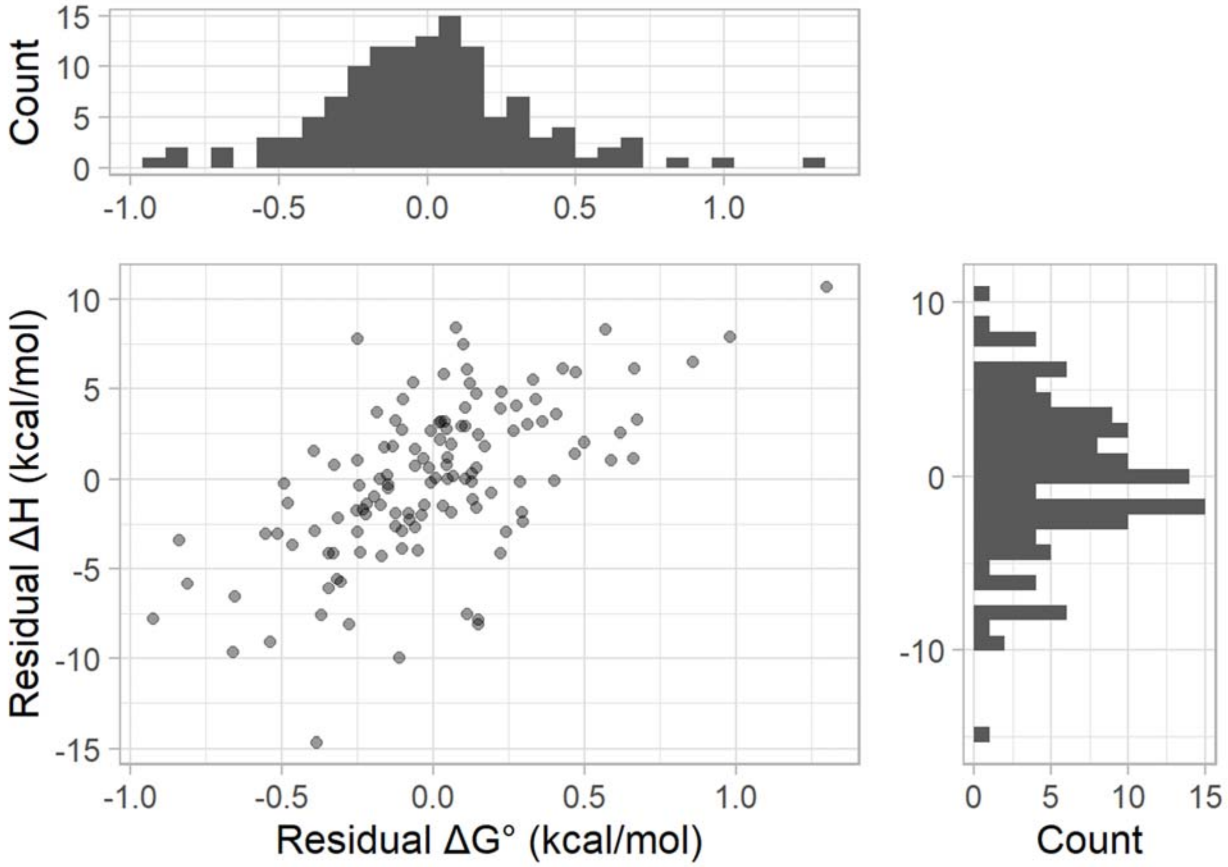

Supplemental Figure 3: Residuals for the Watson-Crick-Franklin stacking parameter predictions. Shown are the scatter plot between the residuals in ΔH° and folding $\Delta G°_{37}$ change predictions (main plot), a histogram of the ΔH° residuals (right plot), and a histogram of the $\Delta G°_{37}$.residuals (top plot).

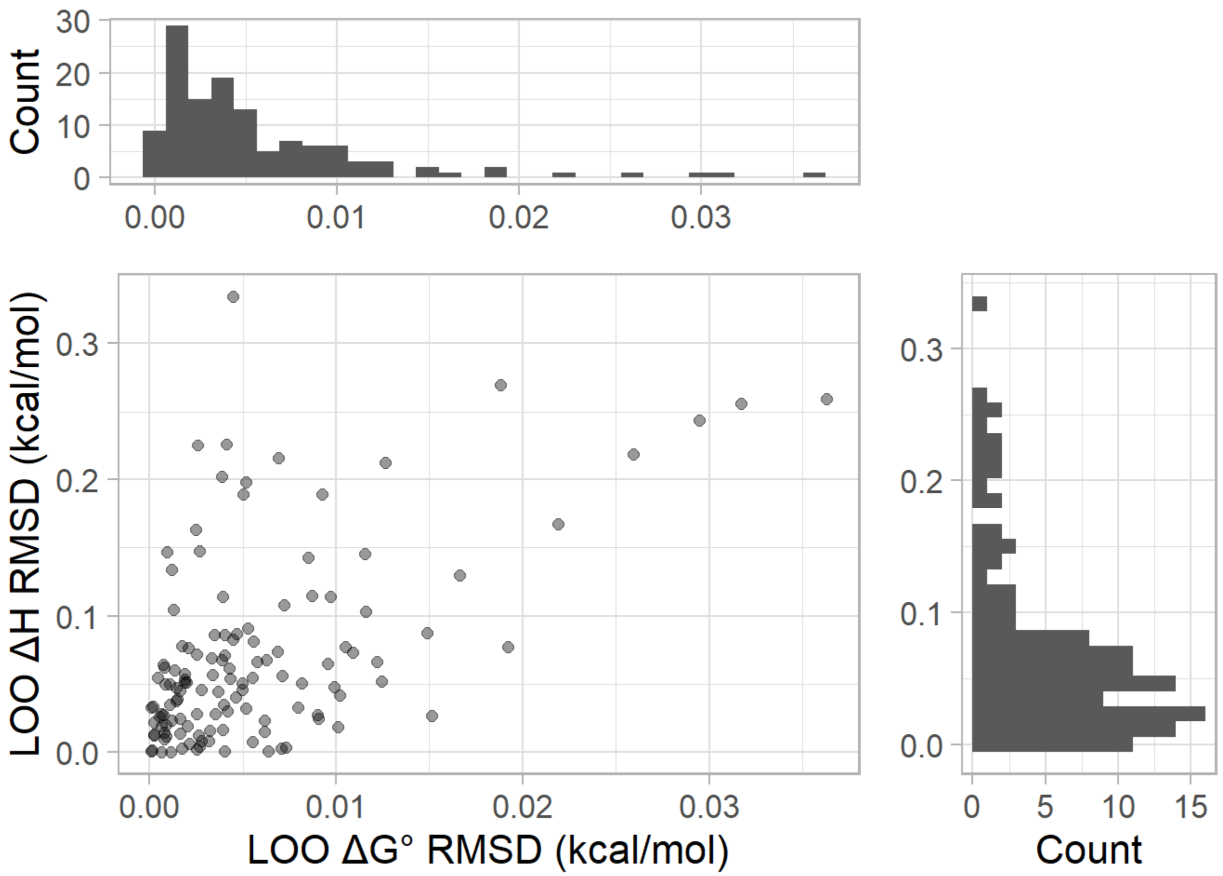

Supplemental Figure 4: Leave-One-Out (LOO) analysis for WCF helix parameters. The plotted value is the RMSD of the parameter values for leaving out a single experiment as compared to when all experiments are used.

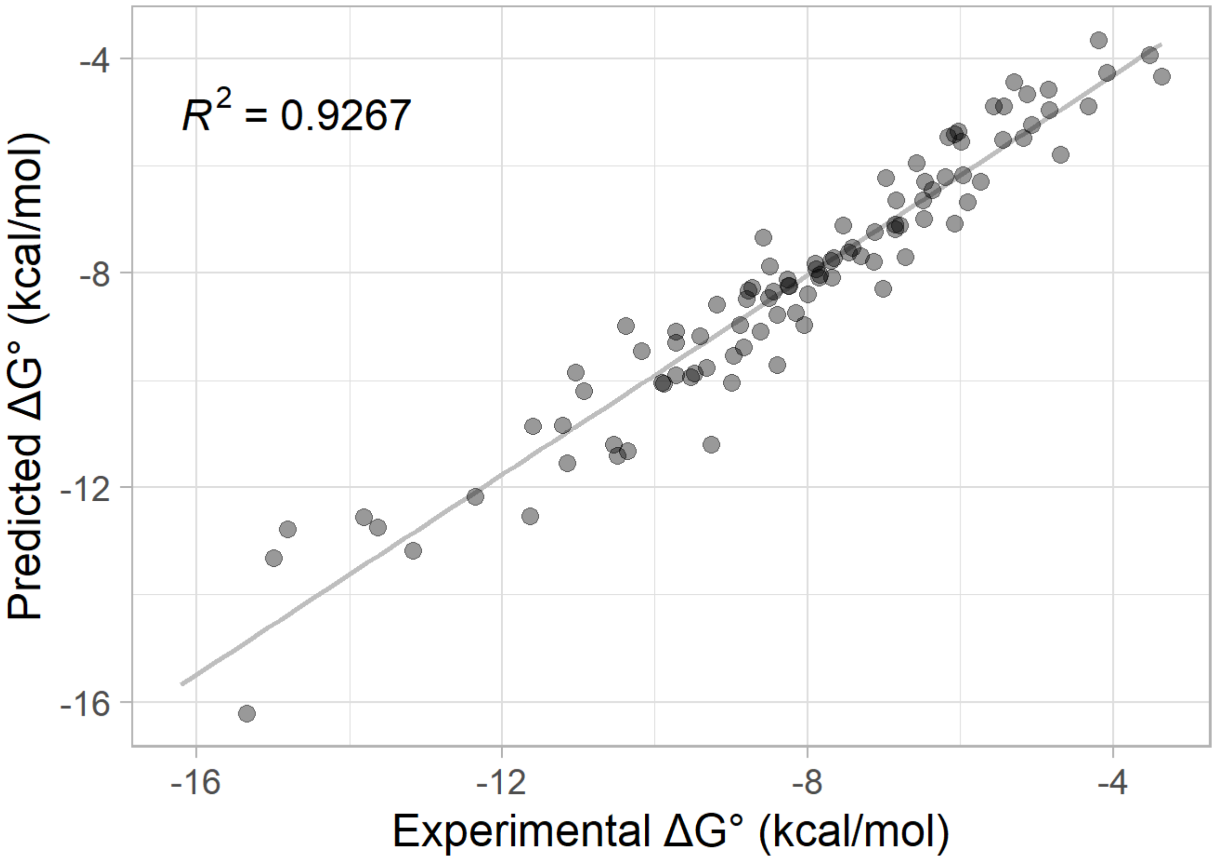

Supplemental Figure 5: Correlation between predicted and observed ΔG°_{37} for duplexes with WCF and GU pairs using a model that includes a $\frac{5'GGUC}{3'CUGG}$ parameter. ΔG°_{37} values predicted from using a model identical to that reported in Table 1 except for the addition of a $\frac{5'GGUC}{3'CUGG}$ parameter are plotted against values determined from optical melting experiments.

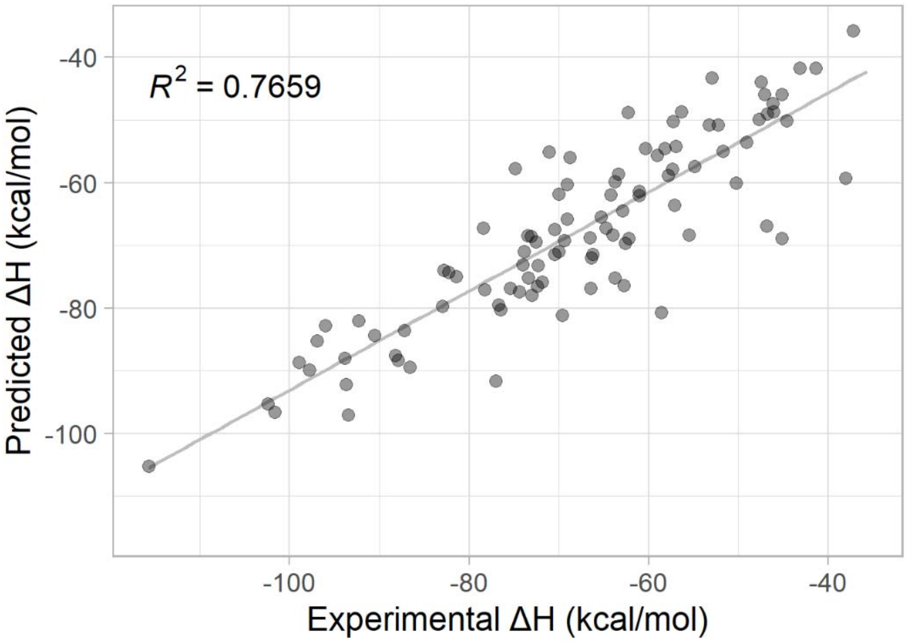

Supplemental Figure 6: Correlation between predicted and observed ΔH° for duplexes with WCF and GU pairs. ΔH° values predicted from parameters in Table 1 plotted against values determined from optical melting experiments.

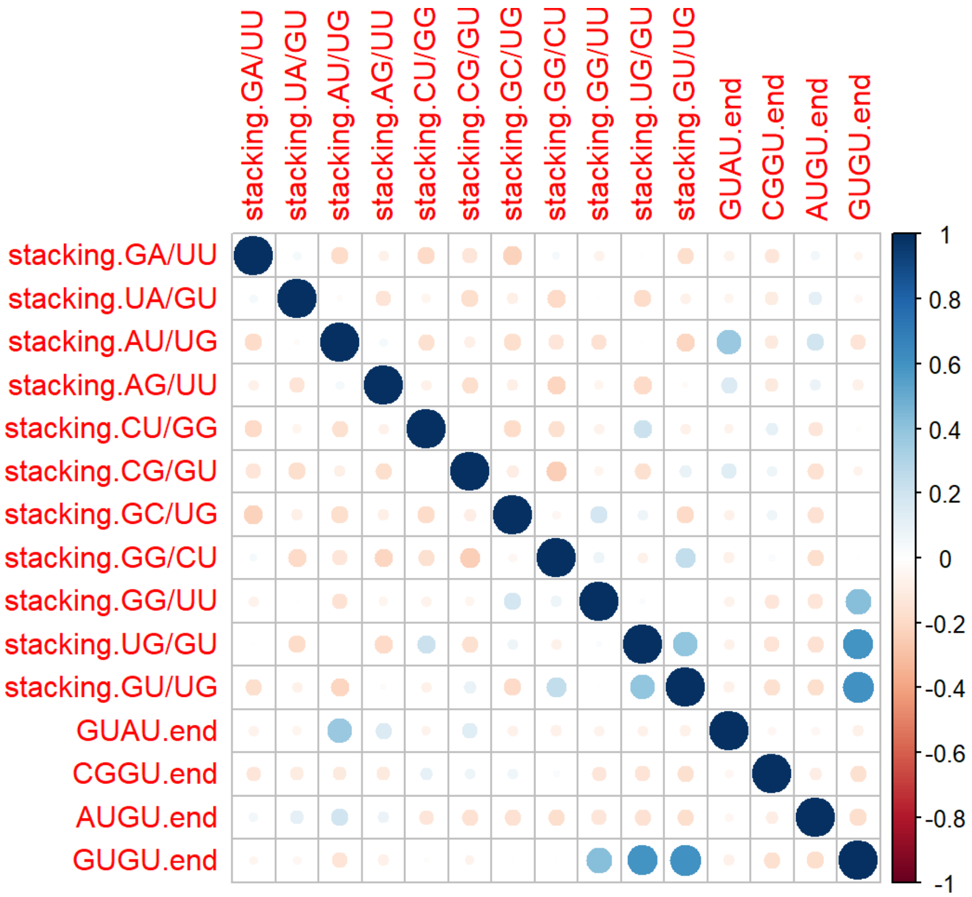

Supplemental Figure 7: Pearson correlation between features of GU stacking model. Pairwise Pearson correlation coefficients were calculated for the observed frequencies of GU stacking parameters. The correlation coefficients are encoded in the size and color of each circle. The base pair stacks are represented a top strand (written 5’ to 3’) that is followed by a bottom strand (written 3’ to 5’). Therefore, “AU/UG” indicates an AU pair followed by a UG pair: $\begin{matrix}5'\text{AU}\\3'\text{UG}\end{matrix}$.

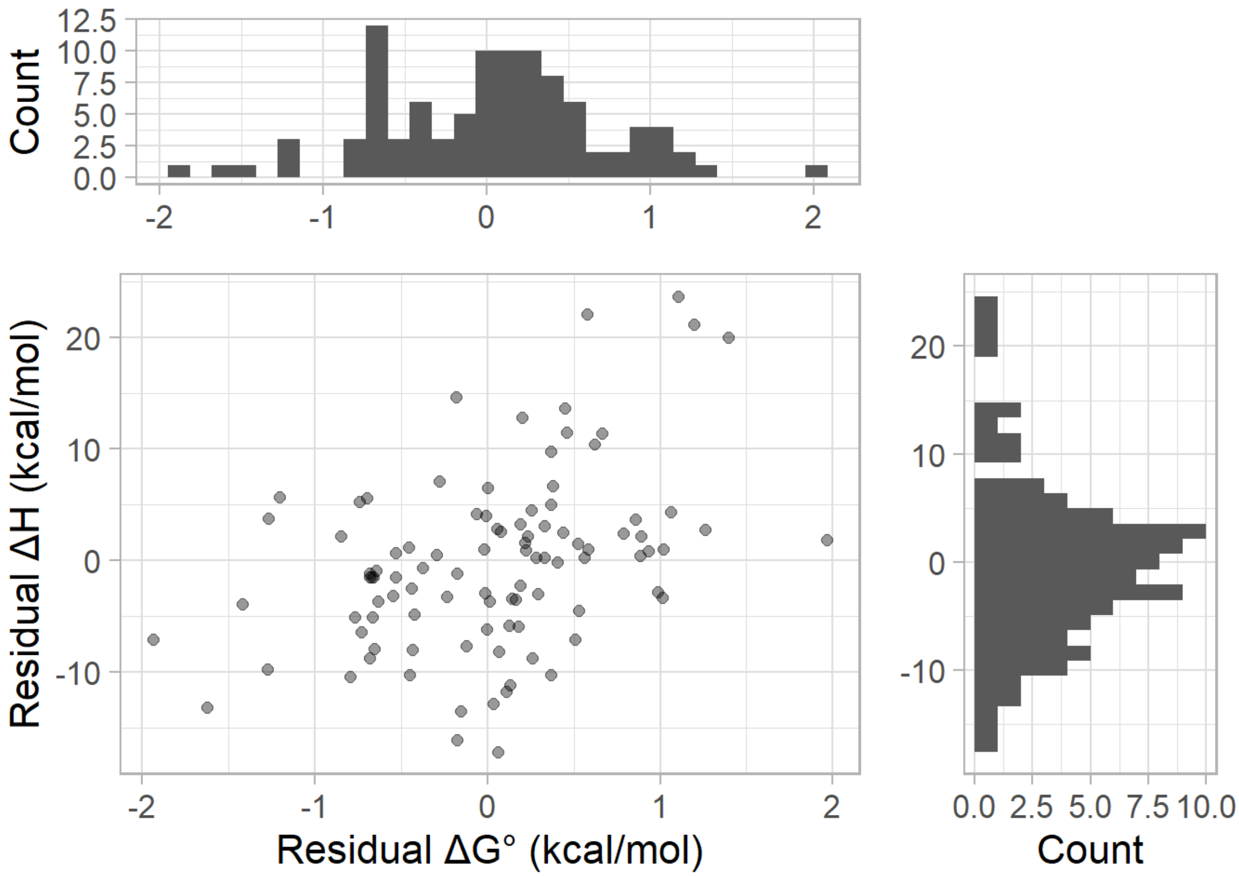

Supplemental Figure 8: Residuals for the GU stacking parameter predictions. Shown are the scatter plot between the residuals in ΔH° and folding ΔG°_{37} predictions (bottom left plot), a histogram of the folding ΔG°_{37} residuals (top plot), and a histogram of the ΔH° residuals right plot).

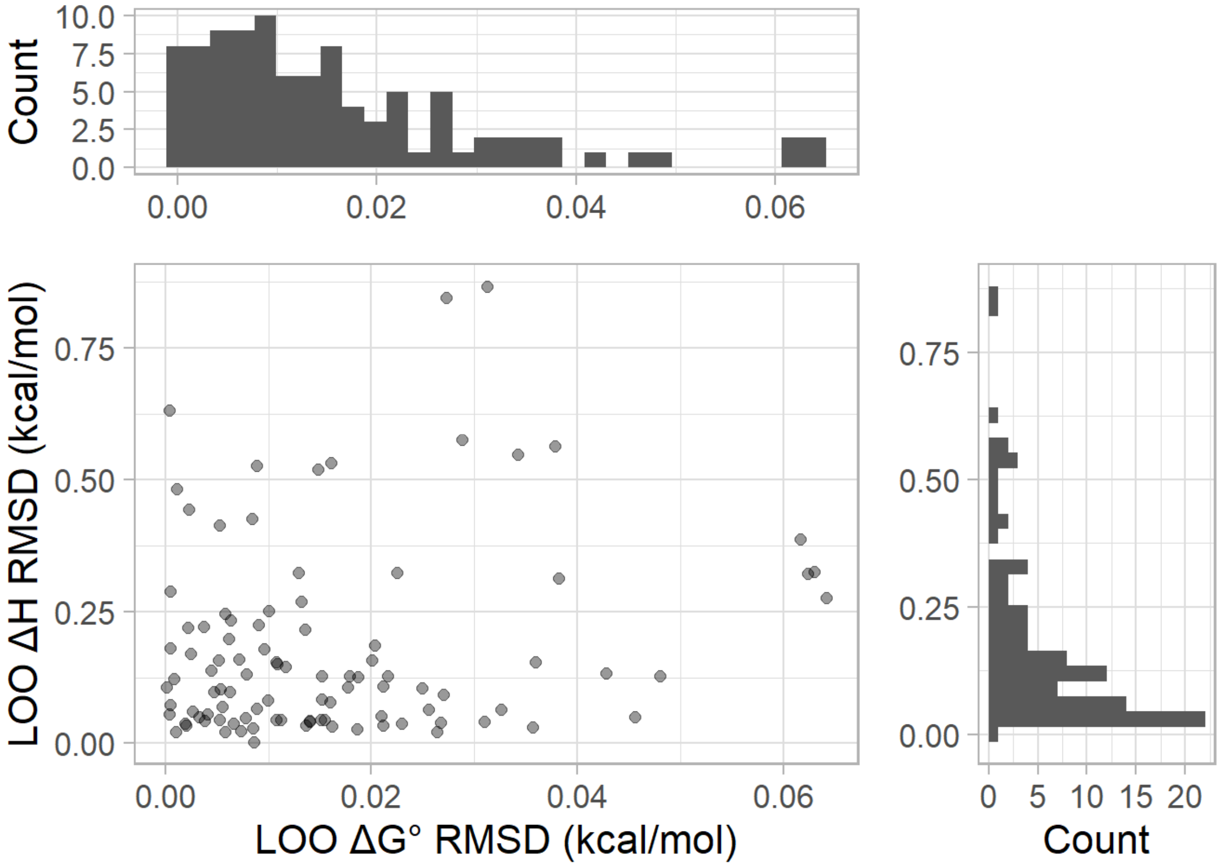

Supplemental Figure 9: Leave-One-Out (LOO) analysis for GU stacking parameters. The plotted value is the RMSD of the parameter values for leaving out a single experiment as compared to when all experiments are used.

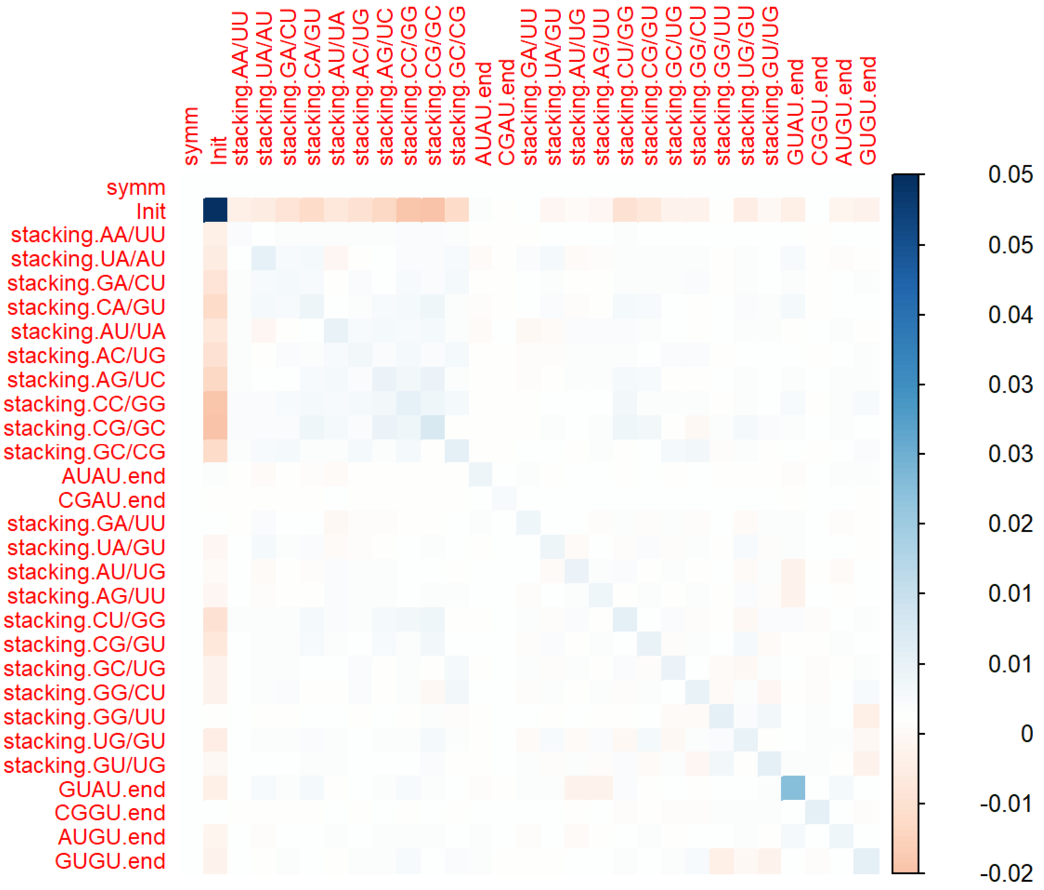

Supplemental Figure 10. The covariation between ΔG°_{37} parameter values. Pairwise covariation values for ΔG°_{37} values fit to 1,000 randomly perturbed sets of optical melting experiments. The base pair stacks are represented a top strand (written 5' to 3') that is followed by a bottom strand (written 3' to 5'). Therefore, "AG/UC" indicates an AU pair followed by a GC pair: $\frac{5'AG}{3'UC}$.

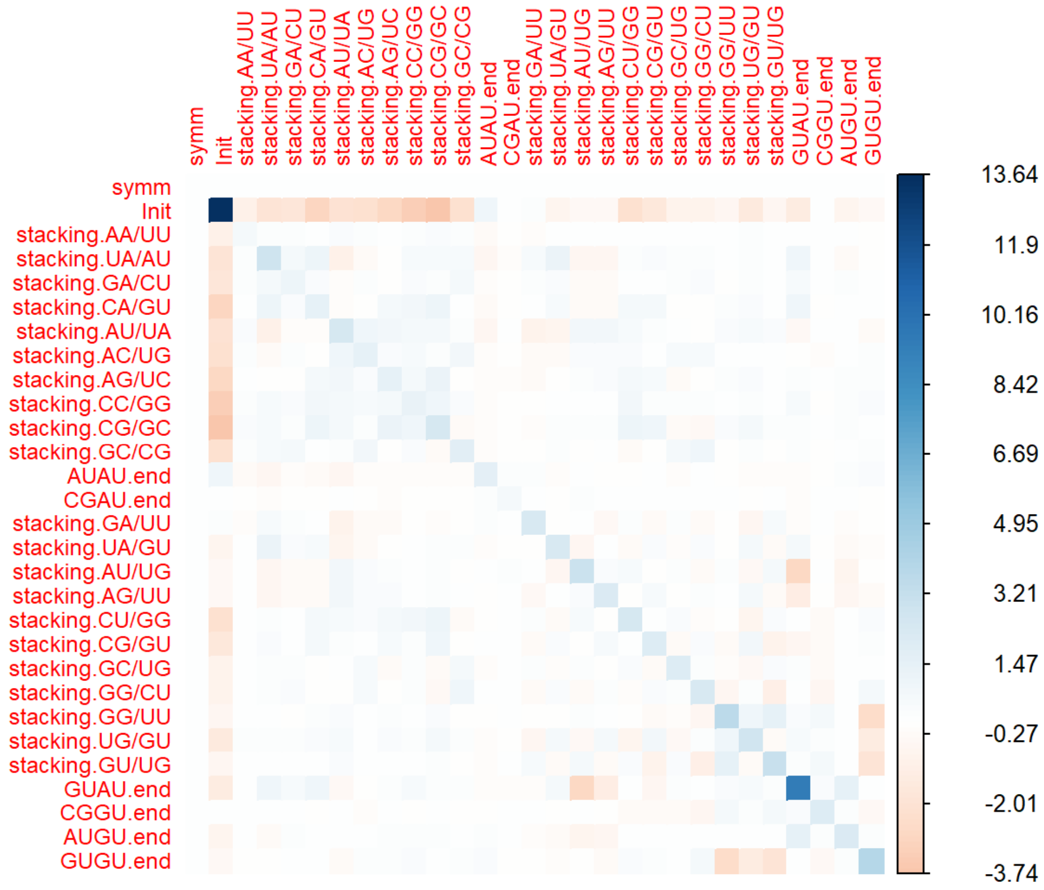

Supplemental Figure 11. The covariation between ΔH° parameter values. Pairwise covariation values for ΔH° values fit to 1,000 randomly perturbed sets of optical melting experiments. The base pair stacks are represented a top strand (written 5' to 3') that is followed by a bottom strand (written 3' to 5'). Therefore, "AG/UC" indicates an AU pair followed by a GC pair: $\begin{matrix}5'AG\\3'UC\end{matrix}$.

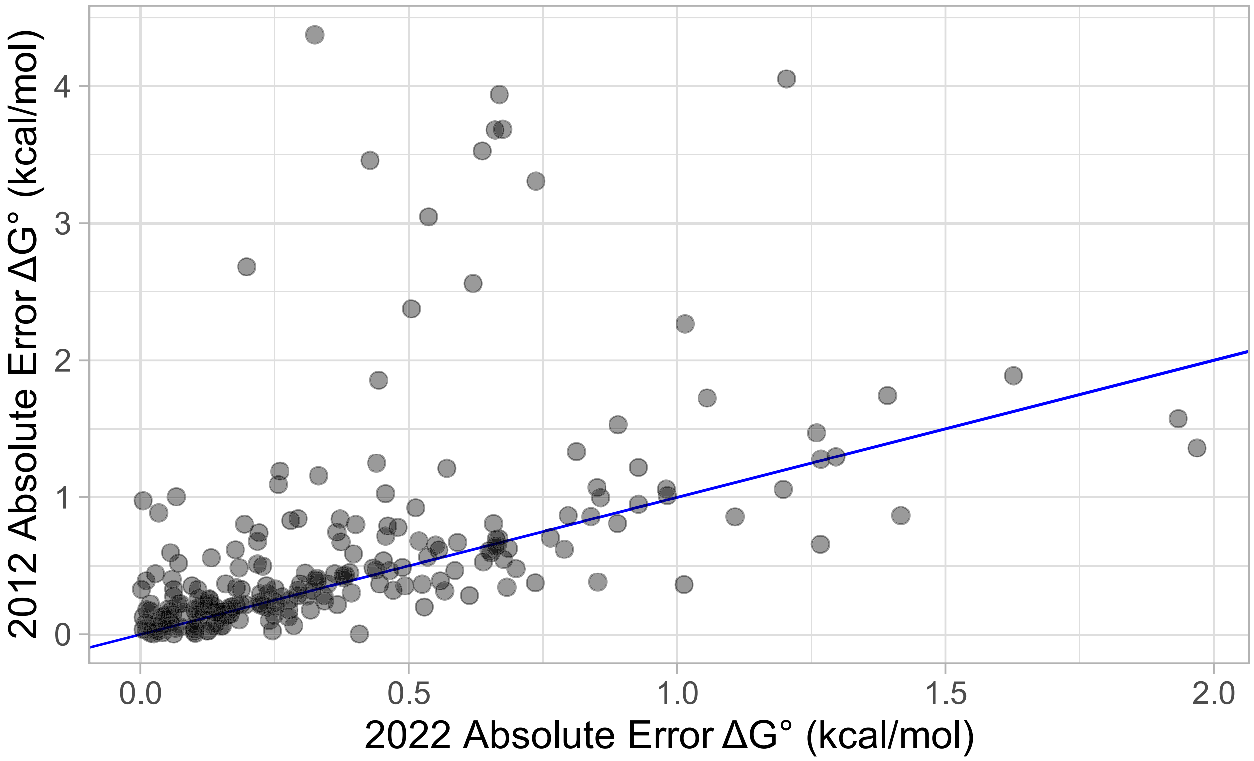

Supplemental Figure 12. The new model improves the fit compared to the previous model. This plot shows the absolute value of the error of the fit (the residual) of ΔG° for the 2012 model as a function of the absolute value of the error of the fit for the 2022 model. All duplexes from Supplemental Table 1 (WCF only and duplexes with GU pairs) are included. The blue line shows the diagonal for reference. A subset of estimates for the 2012 model is substantially improved with the 2022 model. This subset is the set of points to the upper-left, with relatively large errors in the 2012 model and relatively small errors in the 2022 model.

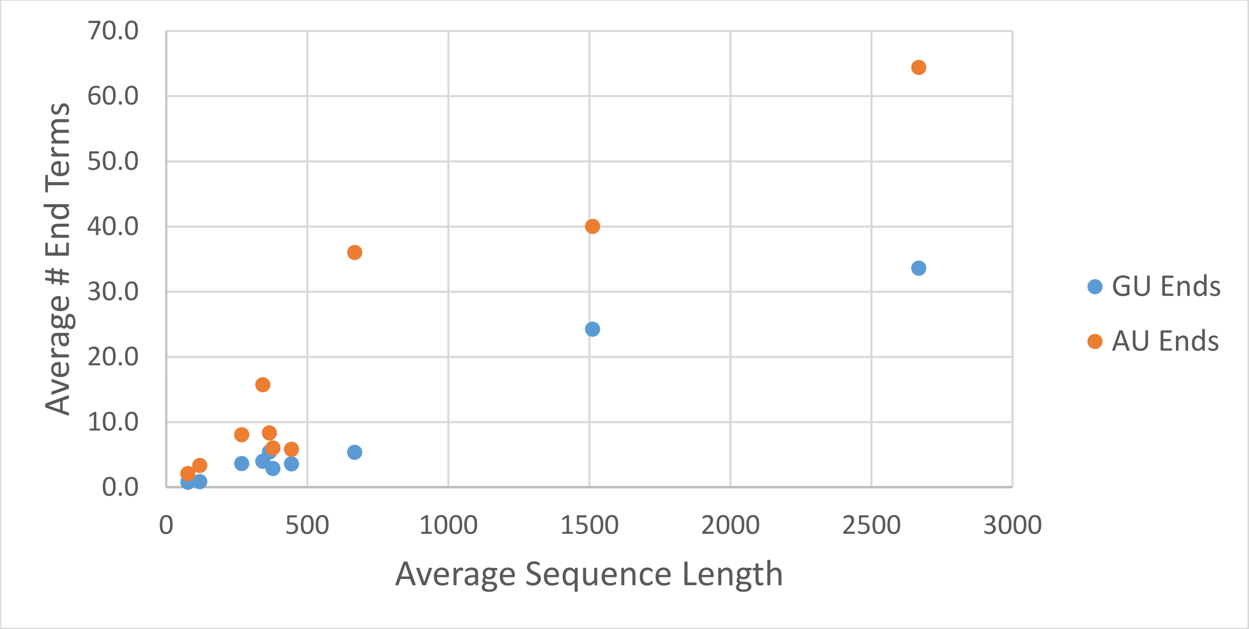

Supplemental Figure 13: AU and GU helix end counts across RNA families. The accepted secondary structures of RNA sequences in different RNA families were parsed to count the number of AU and GU helix ends that existed in the structures. The RNA families included 5S rRNA, 16S rRNA, 23S rRNA, Group 1 and Group 2 introns, RNAP, SRP, telomerase, tmRNA, and tRNA. AU and GU ends were counted if they closed exterior loops, interior loops, hairpin loops, multibranch loops, or bulge loops larger than a single nucleotide.
